# Predisposing factors for bacterial vaginosis, treatment efficacy and pregnancy outcome among term deliveries; results from a preterm delivery study

**DOI:** 10.1186/1472-6874-7-20

**Published:** 2007-10-22

**Authors:** P-G Larsson, Lars Fåhraeus, Bodil Carlsson, Tell Jakobsson, Urban Forsum

**Affiliations:** 1 Department of Obstetrics and Gynecology Kärnsjukuset Skövde, Sweden; 2 Department of Molecular and Clinical Medicine, Division of Obstetrics and Gynecology Linköping University, Sweden; 3 Department of Molecular and Clinical Medicine, Division of Clinical Microbiology, Linköping University, Sweden

## Abstract

**Background:**

Bacterial vaginosis (BV) during pregnancy is associated with an increased risk of preterm delivery but little is known about factors that could predict BV. We have analyzed if it is possible to identify a category of pregnant women that should be screened for BV, and if BV would alter the pregnancy outcome at term; we have also studied the treatment efficacy of clindamycin.

**Methods:**

Prospective BV screening and treatment study of 9025 women in a geographically defined region in southeast Sweden. BV was defined as a modified Nugent score of 6 and above. Data was collected from the Swedish Medical Birth Register. Women allocated to treatment were supplied with vaginal clindamycin cream. The main outcome goals were to identify factors that could predict BV.

**Results:**

Vaginal smears were consistent with BV criteria in 9.3%. Logistic regression indicates a significant correlation between smoking and BV (p < 0.001) and a greater prevalence of BV in the lower age groups (p < 0.001). We found no correlation between BV and history of preterm deliveries, previous miscarriages, extra-uterine pregnancies, infertility problems or reported history of urinary tract infections–factors that earlier have been associated with BV. Treatment with clindamycin cream showed a cure rate of 77%. Less than 1% of women with a normal vaginal smear in early pregnancy will develop BV during the pregnancy. There was no association between BV and the obstetric outcome among women who delivered at term. Women with BV, both treated patients and nontreated, had the same obstetric outcome at term as women with normal vaginal flora.

**Conclusion:**

BV is more than twice as common among smokers, and there is a higher prevalence in the younger age group. However these two markers for BV do not suffice as a tool for screening, and considering the lack of other risk factors associated with BV, screening of all pregnant women might be a strategy to follow in a program intended to reduce the number of preterm births.

## Background

Bacterial vaginosis (BV) is one of the most common vaginal infections with prevalence among pregnant women between 10 and 20% [1-3]. There is an association between BV and preterm delivery [1,2,4-6] and also between BV and early spontaneous miscarriage prior to 16 weeks gestation [7]. Associations between BV and urinary tract infections (UTI) [8,9] as well as between BV and history of infertility caused by tubal factors [10] have been reported in other studies. There is also an association between smoking and BV [11-14].

The primary outcome is to determine the presence of predisposing factors which might be identified at the first antenatal visit and thus reduce the number of women potentially at risk for BV requiring treatment. The secondary outcome were the efficacy of clindamycin treatment during pregnancy and if there are any other associations on the obstetric outcome among term deliveries and BV.

## Methods

All gravida who were booked for antenatal care at clinics in southeast Sweden between February 1999 and May 2001 were invited to take part in an intervention study to investigate whether treatment of BV with clindamycin cream could reduce the frequency of preterm delivery and the morbidity of preterm infants. The result from the latter study has been published [15] and showed that treatment of BV with clindamycin was associated with significant prolongation of gestation with 32 days among women with late miscarriage or preterm delivery. The study at hand is an analysis of the same patient population. A vaginal smear was taken at the first antenatal visit and graded for BV according to Nugent with the exception that a Nugent score of 6 was defined as BV as discussed earlier [15-17].

We used modified Nugent scoring for the diagnosis of BV as we earlier encountered limitations in the use of Nugent scoring. This particularly applies to treatment studies and is even more pronounced when clindamycin vaginal cream is the selected treatment.

At the first antenatal visit, all women were interviewed regarding occupation, civil status, medical history, earlier pregnancies, fertility problems, alcohol, medication, history of UTI, and smoking habits (nonsmokers, smoke 1–9 cigarettes per day or smoke 10 or more cigarettes per day); the latter responses defined smoking habits 3 months prior to conception and the prevailing situation at the first antenatal visit. This information was registered in the antenatal medical record. After delivery, all data collected during the pregnancy, delivery and early puerperal period was sent to the Medical Birth Register (MBR) at the Swedish National Board of Health and Welfare. The study compared MBR data with the status of the vaginal smears. However if a gravida had a termination of pregnancy or a spontaneous abortion prior to 22 weeks gestation, or a intrauterine death between 22–28 weeks gestation no such data is available from MBR.

The women participated in a randomized consent design treatment study according to Zelen [18] which investigated the outcome of treatment with vaginal clindamycin, or alternatively no treatment, on early preterm birth. All women with BV were randomized to either an intervention group with a 7-day regimen of treatment with clindamycin vaginal cream or a control group to remain untreated and uninformed of their BV status as stipulated in the pre-randomized consent design for clinical trials. Only women who were diagnosed with BV and randomized to the intervention group were informed of the status of their vaginal smear. Data from the frequency of preterm delivery is published [15]. Evaluation of treatment efficacy was done with new Gram-stained smears both 12 and 20 weeks after treatment. The treatment results were classified cured, improved, or failure. Cured meant the smear had a Nugent score of < 4 or a Hay/Ison score of 1 [2,19], improved meant the Nugent score was 4–5 or Hay/Ison score was 2, and treatment failure indicated that the Nugent score was ≥ 6 or Hay/Ison score was 3. The women from the randomized control study are included in the analysis.

The study has been approved by the Regional Ethics Committee in Linköping, Sweden and the Medical Products Agency in Sweden.

### Statistics

Statistical analysis with Chi-square test, Odds Ratio (OR) with 95% Confidence Interval (CI) and logistic regression were done using the SPSS program for Windows version 14.0.

## Results

**Figure 1:**
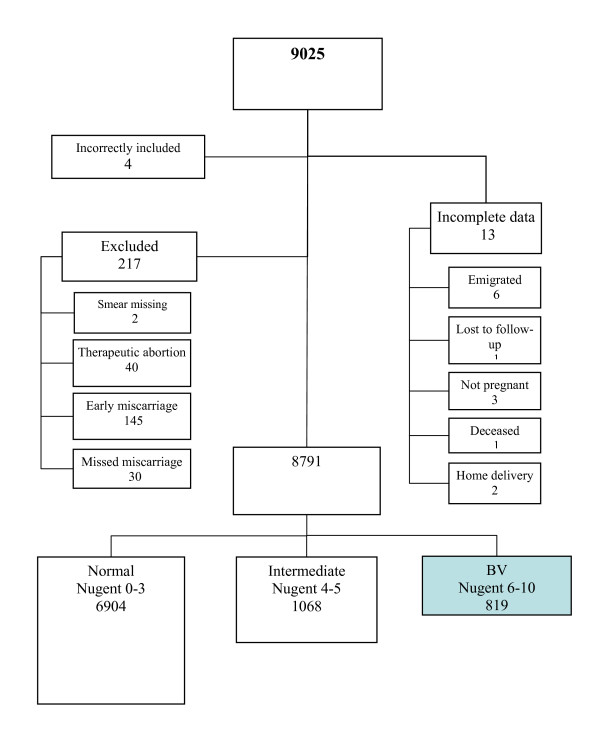
Flow chart of the participation status in the preterm delivery study. At start there were 9025 women and 8791 remained throughout the study.

We analyzed the same patient population that was used in our treatment study [15], thus a total of 9025 women were screened for BV during their first antenatal visit. After exclusion of women who were lost or had terminated pregnancies, the study comprised 8791 women (Figure 1).

**Figure 2:**
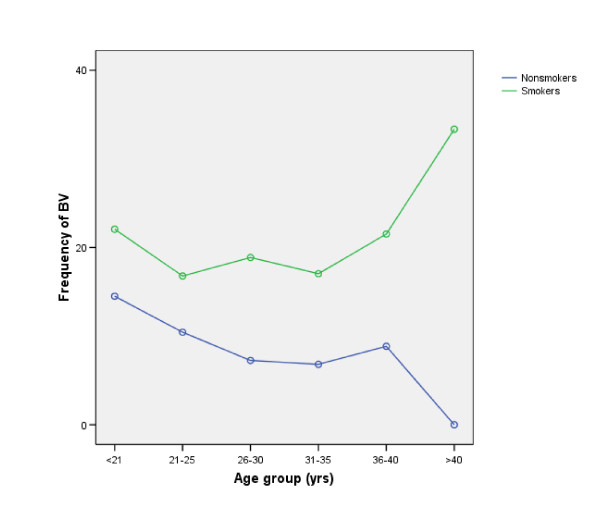
There is a significant declining frequency of BV with increasingage among nonsmokers. (OR for each age group are 0.83 (95% CI 0.75–0.90) but among smokers, the OR are 1.03 (95% CI 0.89–1.20). Women >40 yrs. of age who are smokers have a high percentage of BV, but this age group has a small population (of 9 women, only were 3 smokers).

Evaluation of Gram-stained vaginal smears revealed a prevalence of BV of 9.3%. A higher rate of BV is found in the age group 18–25. The mean age among the women with normal smears was 29.2 versus 28.6 among women with smears indicating BV (p = 0.001, student t-test) (Figure 2).

The percentage of women reporting that they were smokers three months prior to conception was 21.0% (1715/8168) (missing data for 623 women). The percentage of women reporting being smokers at the time of the first antenatal visit was 11.8% (983/8351) (missing data for 440 women). Seven hundred and fifty-nine women stopped smoking when they learned they were pregnant and only nine women took up smoking during early pregnancy.

A greater percentage of smokers are found among women with BV. Three months before pregnancy, 36.4% of the women with BV were smokers compared to 19.4% among the women who had normal vaginal smears (OR 2.4 95% CI 2.0–2.8). Corresponding data supplied at the first antenatal visit indicates 23.4% smokers among BV patients compared to 10.6% among those with normal vaginal smears (OR 2.6 95% CI 2.2–3.1). At the first antenatal visit, the OR was 2.2 to smoke 1–9 cigarettes/day compared to OR 3.0 to smoke ≥ 10 cigarettes/day. (Table 1)

**Table 1:** Correlation between smoking and BV

	BV			No BV			Missing		
	Yes	All BV	%	Yes	All No BV	%			Total
Smoker 3 mos. before 1^st ^visit	278	764	36.4	1437	7404	19.4	623	OR 2.4 (95% CI 2.0–2.8) *	8791
Smoker at 1^st ^antenatal visit	181	774	23.4	802	7577	10.6	440	OR 2.6 (95% CI 2.2–3.1) *	8791
Smoker 1–9 cig/day at 1^st ^visit	116	774	15.0	575	7577	7.6	440	OR 2.2 (95% CI 1.8–2.7)*	8791
Smoker >10 cig/day at 1^st ^visit	65	774	8.4	227	7577	3.0	440	OR 3.0 (95% CI 2.2–3.9) *	8791

* p < 0.05

Women who had stopped smoking just prior to pregnancy or in an early stage (n = 759) had a higher prevalence of BV than those who had never smoked. The prevalence of BV was 18.4% among women who were smokers both before and during early pregnancy, 13.2% in women who had stopped smoking and 7.6% among patients who had never smoked.

We found no correlation between BV in early pregnancy and self-reported miscarriages (19.6% in both groups), infertility more than 1 year before the actual pregnancy, infertility surgery, extra-uterine pregnancies or *in vitro *fertilization. We found no association between BV and diabetes, self-reported history of repeated urinary tract infections (UTI) or maternal underweight.

Of the participating women, 43.5% were primiparous and had BV in 9.7% compared to 8.9% for multiparous women. Multiparous women with BV included in this study did not report a higher frequency of previous preterm birth than women who at inclusion had a normal vaginal flora (7.8% vs. 8.6%). (Table 2)

**Table 2:** Self-reported medical history obtained at the first antenatal visit

	BV			No BV			Missing		
	Yes	All BV	%	Yes	All No BV	%			Total
Earlier miscarriages	156	795	19.6	1534	7821	19.6	175	OR 1.0 (95% CI 0.8–1.2)	8791
Infertility more than 1 year	49	795	6.2	551	7821	7.0	175	OR 0.9 (95% CI 0.6–1.2)	8791
Infertility surgery	2	795	0.3	16	7821	0.2	175	OR 1.2 (95% CI 0.3–5.4)	8791
Extrauterine pregnancy	8	795	1.0	113	7821	1.4	175	OR 0.7 (95% CI 0.3–1.4)	8791
*In vitro *fertilizations	1	795	0.1	54	7821	0.7	175	OR 0.2 (95% CI 0.03–1.3)	8791
UTI	109	795	13.7	1192	7821	15.2	175	OR 0.9 (95% CI 0.5–1.5)	8791
Previous preterm birth	34	402	8.5	383	4444	8.6	0	OR 0.9 (95% CI 0.6–1.3)	4846
Diabetes	1	795	0.1	18	7821	0.2	175	OR 0.5 (95% CI 0.1–4.0)	8791
BMI underweight	17	752	2.3	145	7321	2.0	718	OR 1,1 (95% CI 0.7–1.9)	8791

Binary multiple logistic regression analysis showed significant correlation between BV and smoking (OR 2.53 95% CI 2.11–3.04, p < 0.0001) and BV and women in the younger age groups, i.e. under 26, (OR 0.97 95% CI 0.96–0.99, p < 0.001), even though both smoking and BV are more common in the younger age groups (Figure 2). The frequency of BV among nonsmokers is 14.5% in the age group 18–21, compared to 8.0% in the age group >35.

A total of 408 women were randomized to treatment (with clindamycin cream) at a mean gestational week of 12+ 6, and of these 332 contributed a second smear at a mean time of 12.5 weeks later (gestational week 25+ 3). There is a 15% difference in cure rate depending on the classification used: Nugent score [16] or Hay/Ison [2,19]. According to Nugent scoring there were 63% cured and 22% improved and 15.4% treatment failure, whereas according to Hay/Ison 77% were cured, 12% improved, and 10% treatment failures.

A total of 463 women not receiving treatment were followed-up with a second smear after 10–12 weeks (at the mean gestational week 24). Of the 443 women who did not have BV at the first antenatal visit, only 4 (0.9%) had developed BV during the pregnancy.

Analysis of the outcome of the pregnancies did not show any association between BV and poor obstetric outcome nor did we find any association to instrument or Cesarean delivery, preeclampsia, or early rupture of the membranes whether or not any treatment with clindamycin was given. There was no association between the presence of BV and infant characteristics such as gender, small for gestational age (SGA) or asphyxia. Treatment of BV did not increase the risk for having preeclampsia (OR 1.1 95% CI 0.6–2.2). Of 392 treated women, 4.2 percent had preeclampsia compared to 4.6% of the non-treated women (Table 3).

**Table 3:** Obstetrical outcome among BV patients after treatment with clindamycin

Obstetric outcome		BV			No BV			Missing		
	Effect of treatment	Yes	All BV	%	Yes	All No BV	%			Total
Instrument delivery		64	816	7.8	631	7945	7.9	30	OR 1.0 (95% CI 0.8–1.3)	8791
	Treated BV vs. not treated BV	34	407	8.4					OR 0.9 (95% CI 0.6–1.3)	816
Cesarean delivery		107	816	13.1	1124	7945	14.1	30	OR 0.9 (95% CI 0.7–1.1)	8791
	Treated BV vs. not treated BV	52	407	12.8					OR 0.9 (95% CI 0.7–1.3)	816
Preeclampsia		36	816	4.4	304	7945	3.8	30	OR 1.2 (95% CI 0.8–1.7)	8791
	Treated BV vs. not treated BV	17	407	4.2					OR 1.2 (95% CI 0.7–1.9)	816
Early rupture of membranes		43	816	5.3	391	7945	4.9	30	OR 1.1 (95% CI 0.8–1.5)	8791
	Treated BV vs. not treated BV	24	407	5.9					OR 0.9 (95% CI 0.6–1.5)	816
SGA		49	815	6.0	374	7920	4.7	56	OR 1.3 (95% CI 0.95–1.8)	8791
	Treated BV vs. not treated BV	21	407	5.2					OR 1.4 (95% CI 0.98–2.2)	815
5 min.		22	819	2.7	191	7972	2.4	0	OR 1.1 (95% CI 0.7–1.8)	8791
	Treated BV vs. not treated BV	7	407	1.7					OR 1.5 (95% CI 0.9–2.6)	819
Gender = Female		379	815	46.5	3846	7933	48.5	43	OR 0.9 (95% CI 0.8–1.1)	8791

## Discussion

This study of factors that predict BV during pregnancy is one of the most comprehensive ever published and is based on around 9000 pregnant women. We found that smoking is strongly associated with BV as indicated by at least a doubled prevalence of BV among smokers as compared to nonsmokers. This has been shown by others [11-14] and there are several possible explanations. The first is that smoking does indeed have a causal connection to BV, the second is that women who smoke may have a risk behavior that would predispose to BV, and the third is that women who smoke may not notice the malodor caused by BV. As we could demonstrate that BV in early pregnancy is more common among women who have stopped smoking compared to women who had never smoked, there might be a causal connection between BV and smoking. Nicotine in the vagina/cervix could have a negative impact on vaginal flora. To our knowledge, our study is the first to indicate that BV is more common among women who are former smokers than among those who have never smoked.

We also found that BV did not increase with increasing age as suggested earlier [20] a supposition which is very commonly quoted in reviews of BV. The frequency of BV in our study was 16% in the age group 18–21 years compared to 8% in the age group 31–35 years (Fig 2). The often quoted studies come from different populations such as sexually transmitted disease clinics. The fact that BV prevalence increases with age has been used as an argument that BV is not a sexually transmitted infection (STI) as other STIs are more common among younger women [21].

We used modified Nugent scoring for the diagnosis of BV [15,17] as we earlier encountered limitations in the use of Nugent scoring. This particularly applies to treatment studies and is even more pronounced when clindamycin vaginal cream is the selected treatment. Clindamycin vaginal cream eradicates almost all bacteria in the vagina, and greatly reducing the lactobacilli morphotype bacteria and giving the Nugent score of around 4. This means that the smear can be incorrectly interpreted as demonstrating intermediate flora. On the other hand if there are only five gardnerella morphotype bacteria per vision field, the Nugent score would increase from 0 to 3 even when there are more than 500 lactobacilli morphotype bacteria [17]. The Hay/Ison [2,19] classification is better adapted to follow-up treatment results, and for this reason we have deliberately expressed the efficacy in two different ways showing a 15% difference in efficacy depending on the evaluation method used. This has to be taken into consideration when discussing treatment efficacy.

Our study also indicates that there is a less than 1% risk of developing BV among women whose early screening shows normal lactobacilli flora. A similar finding has been reported earlier [22,23].

Therefore we conclude that one BV screening in early pregnancy will suffice to identify women with increased risk for BV and thus also preterm birth.

Despite the comprehensive scope of this study, we could not demonstrate any correlation between the presence of BV and previous miscarriage, infertility, extra-uterine pregnancies or recurrent UTI, factors that other studies have associated with BV [8-10]. We found no association to adverse pregnancy outcome between the women with normal lactobacilli or BV. Ugwumadu et al. found a nonsignificant trend for an association between pre-eclampsia and treatment with clindamycin [4] but we could not verify this even though we had 340 preeclampsia patients in our material. Only women who delivered were included in our study, otherwise no supplementary data would be available from MBR. The fact that we studied only pregnant women might be a selection bias since infertile women were not included. Ralph et al. reported that BV during pregnancy predisposed to an increased rate of very early miscarriages after assisted fertilization [24], a circumstance we can not demonstrate in our study, and this might explain why we found no correlation between BV and earlier miscarriages. Ours is the first study to show that there are no increased risks for poor obstetrical outcome in term pregnancies even if the women are infected by BV.

## Conclusion

The conclusion from this study is that there are no factors, based on the interview answers given at the first antenatal visit, that could predict the presence of BV and that selective screening based on this type of data gathering is not possible. The only alternative would be to screen all pregnant women. Smoking habits influence the risk for women to acquire BV. As both smoking and BV in turn increase the risk for late miscarriage, preterm rupture of the membranes, and preterm delivery, both factors (smoking and BV) ideally should be eliminated during pregnancy. To screen for BV, only one vaginal sample is required, and if this is consistent with normal vaginal flora, there is no need to take another smear during pregnancy as the risk of developing BV is less than 1%.

## List of abbreviations used

Bacterial vaginosis (BV)

Body mass index (BMI)

Confidence Interval (CI)

Medical Birth Register (MBR) at the Swedish National Board of Health and Welfare

Odds Ratio (OR)

Small for gestational age (SGA)

Urinary tract infections (UTI)

## Competing interests

P-G.L. has received payment for lectures; also funding for trials from Pharmacia Ltd now Pfizer Ltd. All other authors declare that they have no competing interests

## Authors' contributions

P-GL, LF, TJ, BC and UF contributed to design and collection of data, P-GL, LF and UF contributed to interpretation of results, statistical analysis and report writing. All authors have read and approved the final manuscript

## Pre-publication history

The pre-publication history for this paper can be accessed here:


